# Psychometric properties of the EQ-5D-5L in diabetes mellitus patients in Spain

**DOI:** 10.1186/s41687-025-00874-5

**Published:** 2025-05-29

**Authors:** Amaia Bilbao-González, Marta González-Sáenz de Tejada, Montse Ferrer, Yolanda Ramallo-Fariña, Miguel Paja-Fano, Carlos García-Forero, Daniela Mestre, Iñigo Gorostiza-Hormaetxe

**Affiliations:** 1 Osakidetza Basque Health Service, Research and Innovation Unit, Basurto University Hospital, Bilbao, Spain; 2 Network for Research on Chronicity, Primary Care, and Health Promotion (RICAPPS), Bilbao, Spain; 3 Biosistemak Institute for Health Systems Research, Bilbao, Spain; 4 Department of Medicine, Faculty of Health Sciences, University of Deusto, Bilbao, Spain; 5 Biobizkaia Health Research Institute, Barakaldo, Spain; 6 Health Services Research Group, IMIM (Hospital del Mar Medical Research Institute), Barcelona, Spain; 7 CIBER de Epidemiología y Salud Pública, CIBERESP, Madrid, Spain; 8 Universitat Pompeu Fabra (UPF), Barcelona, Spain; 9 Canary Islands Health Research Institute Foundation, Tenerife, Spain; 10 Osakidetza Basque Health Service, Department of Endocrinology, Basurto University Hospital, Bilbao, Spain; 11 Faculty of Medicine, University of the Basque Country UPV/EHU, Leioa, Spain; 12 School of Medicine, Universitat Internacional de Catalunya, Sant Cugat del Vallès, Barcelona, Spain

**Keywords:** Diabetes mellitus, EQ-5D-5L, Utility index, Psychometric properties, Item response theory

## Abstract

**Background:**

The EQ-5D-5L five-dimensional instrument, is one of the most widely used generic preference-based questionnaires to measure health-related quality of life and to estimate utility indices for use in economic evaluation. This study aimed to assess the psychometric properties of the Spanish EQ-5D-5L questionnaire in patients with Diabetes Mellitus (DM) assessing reliability, validity, and item-level properties such as item functioning.

**Methodology:**

We included 133 patients with DM who completed the EQ-5D-5L, the Audit on Diabetes-Dependent Quality of Life (ADDQoL), the Hospital Anxiety and Depression Scale (HADS), one question about general health and sociodemographic, and clinical data. The reliability was assessed by Cronbach’s alpha, and the item functioning by the item response theory (IRT). Convergent validity was tested using the Spearman correlation coefficient between EQ-5D-5L, ADDQoL, HADS and the general health question. We examined known-groups validity by comparing the EQ-5D-5L scores between subgroups defined by age, gender, BMI, regular physical activity, disease duration, glycemic control by glycosylated blood hemoglobin (HbA1c) (%), type of DM, general health and anxiety and depression level using *t*-test, ANOVA, Wilcoxon or Kruskal-Wallis tests.

**Results:**

The reliability was supported with a Cronbach’s alpha of 0.78. The IRT results supported the unidimensionality and showed adequate item functioning, except for the anxiety/depression dimension. The item with highest discriminatory power was usual activities dimension, followed by self-care and mobility dimensions. The EQ-5D-5L showed adequate convergent validity, with high correlation with the ADDQoL, HADS and general health. Older age, women, obese, no regular physical activity, ≥ 10 years of disease duration, poor glycemic control, poorer general health and higher anxiety and depression level linked with lower EQ-5D-5L scores.

**Conclusions:**

These findings support the adequate psychometric properties of the EQ-5D-5L in patients with DM, supporting its use for clinicians and researchers as an outcome measure and for use in economic evaluation studies.

## Background

Diabetes mellitus (DM) is one of the most important public health problems worldwide due to its high prevalence, morbidity, influence on patients’ health related quality of life (HRQOL) and impact on the healthcare system [1]. In Spain, the age- and sex-adjusted prevalence of DM is 13.8%, and the prevalence of undiagnosed diabetes is 6% [2]. In the long term, diabetes is associated with vascular complications, episodes of hypoglycemia, lifestyle changes and fear of long-term consequences, which may impair HRQOL [3]. This leads to a higher demand for medical and patient care and higher health care costs per patient. In Spain, the total cost of caring for patients with type 2 diabetes was around 2,500 euros per patient per year in 2010 [4].

HRQOL can be defined as a subjective and multidimensional concept that comprises the physical, psychological and social functioning [5]. In developed countries where life expectancy has been steadily increasing, HRQOL outcomes are essential for describing health. It is recommended that HRQOL be measured using specific and generic questionnaires [6]. Generic measures can be preference-based, allowing utilities to be estimated for use in cost-effectiveness studies, and they enable comparisons with the general populations [7, 8].

Cost-effectiveness evidence has become more important than ever for decision-makers at various levels [9]. In these studies, the most commonly used technique has been to estimate the incremental cost per quality-adjusted life years (QALY) [10] gained with new health technologies. The QALY combines life years and quality of life and allows a broad comparison between different treatment strategies, patient populations, and clinical settings. The parameters needed to calculate QALY are the utility for a given health state, and the amount of time spent in that state. The utilities, usually measured with generic questionnaires, represent the strength of a person’s preferences for health states, with values ranging from 0 (dead) to 1 (full health), although scores can also take negative values for states worse than death [10].

As a generic preference-based measure for describing and valuing HRQOL, the EQ-5D is one of the most widely used [11–13], developed by the EuroQol Research Foundation [14]. The standard format of the EQ-5D consists of five dimensions of health, each with three response options (EQ-5D-3L). There is extensive literature to support the validity and reliability of this 3-level version in many conditions and populations [14]. Nonetheless, its restricted ability to discriminate between different levels of health [15, 16], presence of ceiling effects and skewed distribution have long been recognized [17, 18]. Seeking to overcome these shortcomings, the EQ-5D-5L was developed [12], expanding the range of responses to each dimension from three to five levels. Previous studies have indicated that the 5L version improves upon the 3L version in measurement properties with smaller ceiling effects and a better ability to discriminate between different levels of health [19–22].

Due to the extensive use of EQ-5D-5L in Spanish patients with DM for HRQoL assessing in research, clinical practice and health economics [23–25], it must be ascertained that EQ-5D-5L is a valid and reliable tool specifically in this population. In other words, it is a requirement proving that EQ-5D-5L is a useful tool that accurately and consistently measures the HRQOL in Spanish DM patients. Several studies have evaluated the psychometric properties of the EQ-5D-5L in patients with DM in other cultures and languages, which accumulates evidence about its validity in those cohorts [26–32]. Nonetheless, to the best of our knowledge, it has yet to be validated in this population in Spain. Moreover, no studies have explored its item-level properties assessing item functioning in addition to performing classical validation.

The aim of the current study was to carry out a comprehensive study of the psychometric properties of the Spanish version of the EQ-5D-5L questionnaire in patients with DM investigating its reliability and validity, as well as the item-level properties assessing item functioning.

## Methods

### Study population

We included patients from two hospitals in the Basque Country between July 2013 to December 2015 with an ICD-9-CM diagnosis code of 250. All patients were given a letter informing them about the study and inviting them to participate during a DM follow-up appointment. Patients were excluded if they had a terminal illness, or psychiatric and/or sensory disturbances that could prevent them from answering the questionnaires, as well as if they did not provide informed consent. The institutional review board of the hospitals approved the study (identification number PI2012156-PI12-01473) on 4rd February 2013.

### Measurements

In the follow-up visit, patients who accepted participating were asked to complete the EQ-5D-5L [12], the Audit on Diabetes-Dependent Quality of Life (ADDQoL) [33], the Hospital Anxiety and Depression Scale (HADS) [34] and one question about general health from the 36-item Short Form Health Survey (SF-36) [35].

In addition, a clinician gathered other data on patients in a case report form created adhoc with information obtained for and during the visit that included: -sociodemographic characteristics, namely, age, gender, employment status, level of education; and–clinical characteristics, namely, body mass index (BMI), disease duration, smoking status, regular physical activity, type of DM, insulin treatment, self-monitoring of blood glucose and glycosylated hemoglobin (HbA1c) (%) like a measure of the glycemic control as recommended by the American [1] and Spanish [36] Diabetes Association. Trained personnel collected comorbidities measured by the Charlson Comorbidity Index [37] from the patients’ medical records.

### HRQOL questionnaires

The EQ-5D-5L [12] contains items concerning five dimensions, mobility, self-care, usual activities, pain/discomfort and anxiety/depression, rated on a five-point scale from 1 (no problems) to 5 (unable to/extreme problems). The combined dimensions describe 5^5^=3,125 theoretically possible states of health that can be converted into a weighted index score. For the Spanish value-set, the index score ranges from − 0.416 to 1, with a higher score indicating better HRQOL [38]. Additionally, the questionnaire includes a visual analogue scale (EQ-VAS) on which individuals rate their own health today on a scale from 0 (worst imaginable health) to 100 (best imaginable health).

The ADDQoL [33] is a specific tool for DM consisting of 21 items, 19 of which refer to specific aspects of life (such as social life and work life) scored on a 5-point scale. The impact of diabetes on each of these 19 specific aspects of life is weighted according to its importance to the patient’s quality of life, yielding an average weighted impact (WI) score. This score ranges from − 9 (maximum negative impact of diabetes) to + 3 (maximum positive impact of diabetes). The other two items are summary items: one measures the impact of diabetes on quality of life (-3 indicating maximum negative impact to + 1 as maximum positive impact) and the other measures the current quality of life (-3 indicating extremely poor and + 3 excellent). We used the already validated Spanish version [39, 40].

The HADS is a self-report measure designed specifically for people with a physical illness [34], which is divided into two subscales, anxiety (seven items) and depression (seven items). Each of the 14 items is rated on a 4-point Likert scale and the total score for each subscale ranges from 0 to 21, with a higher score indicating a higher level of mood disorder. A score ≤ 7 corresponds to “no depression/anxiety”, a score from 8 to 10 corresponds to “minor depression/anxiety”, and a score ≥ 11 indicates “moderate to severe depression/anxiety”. The Spanish version used has been validated [41, 42].

We used the question about general health “In general would you say your health is?” from the SF-36 questionnaire [35], which has five response options as follows: 1 (poor), 2 (fair), 3 (good), 4 (very good) and 5 (excellent).

### Statistical analysis

Data from patients that responded to the whole EQ-5D-5L were used for the analyses. For the descriptive analyses, means and standard deviations (SDs), or frequencies and percentages were used. To describe the EQ-5D-5L scores, we also calculated the median, range and floor and ceiling effects, which should be small (< 15%) [43]. The missing data was also examined.

#### Reliability

We assessed internal consistency using Cronbach´s alpha [44]. A coefficient over 0.70 was considered acceptable [45]. Further, the inter-item correlation and the item-scale correlation correcting for overlap were assessed by calculating Spearman’s correlation coefficient.

#### Item functioning

The item functioning of the EQ-5D-5L was studied using item response theory (IRT), which unlike classical test theory, focuses on item-level rather than scale-level properties [46]. A requirement of IRT is that the scales must be unidimensional. Therefore, we first tested for unidimensionality by exploratory (EFA) and confirmatory (CFA) factor analysis. Regarding EFA, an item was considered to contribute to the factor if the factor loading was ≥ 0.40 [47], and a ratio of the eigenvalues of the first and second unrotated components of at least 3:1 was taken as evidence of unidimensionality [48, 49]. CFA was also performed to confirm that the five items load on a single factor. The maximum likelihood estimator was used, and the following indices were calculated [50, 51]: the root mean square error of approximation (RMSEA), for which a value < 0.08 was considered acceptable; and the non-normed fit index (NNFI) and Comparative Fit Index (CFI), for both of which values > 0.90 were considered acceptable.

Then, having confirmed the unidimensionality, we used an IRT model, specifically, the graded response model (GRM), which is useful in the evaluation of polytomous items [52]. In the GRM, the two common item parameters are the item slope (*α*), which captures the ability of an item to discriminate between people with different levels of the latent trait, and the item difficulty (*β*), also called the threshold, which indicates the point on the scale of the latent trait where a person has 0.5 probability of responding positively to an item category [46]. Therefore, item responses are conceptualized in terms of the slope parameter (*α*), and a series of *k*–1 category thresholds, where *k* is the number of item response options. In this case, each item has five response options, but due to the low frequency of response options 4 and 5, they were merged. Therefore, each item is defined by *α* and three *β* thresholds. The slope *α* is comparable to the factor loading of CFA, with higher values indicating that an item has greater discriminatory power while the *β* thresholds are indicative of the spacing of item responses along the trait dimension [49]. That is, the range of the latent trait that the estimated *β* parameters encompass indicates the adequacy of an item to represent low, medium, or high levels of a trait. We use a Bayesian estimator [53] in the GRM models.

This GRM was applied to the EQ-5D-5L items and the slope (*α*) and thresholds (*β*), as well as standard errors (*SEs*), were calculated [54]. These parameters were used to plot a category response curve (CRC) for each item, which represents the probability of a positive response to each item’s response option as a function of the latent trait [48]. In addition, item information curves (IICs) were generated [49], which identify the position on a given trait spectrum at which the item provides the most information. In these graphs, the *x*-axis represents the latent trait, with a scale standardized to have a mean of 0 and *SD* of 1. The fit of the GRM model was assessed by: (a) determining the residuals of the model, by comparing the observed and expected proportions of each response category of each item; and (b) calculating a likelihood ratio χ^2^ for each item, to assess the goodness-of-fit between the expected and observed frequencies [46, 48, 49].

#### Convergent and discriminant validity

The convergent and discriminant validity was assessed by analyzing the relationship of the EQ-5D-5L with the ADDQoL, HADS, and the general health scores, by using Spearman’s correlation coefficient (*ρ*). We considered a coefficient | *ρ*|≥0.5 high; between 0.3 and 0.5, moderate; and between 0.1 and 0.3, small [55]. We hypothesized that the ADDQoL present HRQOL item would be highly correlated (convergent validity) with all EQ-5D-5L scores. Further, we hypothesized that the correlation of the EQ-5D-5L with the influence of diabetes on HRQOL and the average WI would be moderate or low because they are less directly related to different aspects of patients’ HRQOL (discriminant validity). Because four out of the five dimensions of the EQ-5D-5L (mobility, self-care, usual activities and pain/discomfort) are closely related to physical aspects, we hypothesized that the correlation of these four dimensions, the EQ-5D-5L index and the EQ-VAS with the HADS domains would be moderate or low (discriminant validity), and high with the anxiety/depression dimension of the EQ-5D-5L (convergent validity). Further, we hypothesized that there would be a high correlation between the SF-36 general health question and the EQ-5D-5L index and EQ-VAS (convergent validity), and a moderate correlation with each EQ-5D-5L specific dimension (discriminant validity).

#### Known-groups validity

Known-groups validity of the EQ-5D-5L utility index and EQ-VAS was evaluated by comparing subgroups of patients known to differ in health status [28, 29, 31, 56, 57]: age (age ≤ 49; 50 ≤ age ≤ 59; 60 ≤ age ≤ 69; age ≥ 70 years), gender, BMI (< 30 non-obese vs. ≥ 30 obese), regular physical activity (yes/no), disease duration (< 10 vs. ≥ 10 years), glycemic control (poor HbA1c ≥ 7% vs. optimal HbA1c < 7%), type of diabetes (1 vs. 2), SF-36 general health question (poor; fair; good; very good + excellent), and HADS anxiety and depression level (≤ 7 vs. 8–10 vs. ≥11). We hypothesized that both scores would be lower in patients who were older, women, obese, and smokers, did not do regular physical activity, have a disease duration of ≥ 10 years, poor glycemic control (≥ 7%), diabetes type 2, and had a poorer SF-36 “health today rating” and higher depression or anxiety level. These comparisons were made using a *t*-test or analysis of variance, with Scheffe’s test for multiple comparisons, or the non-parametric Wilcoxon or Kruskal-Wallis tests.

All effects were considered statistically significant at *p* < 0.05. The statistical analyses were performed with SAS 9.4 for Windows (SAS Institute, Cary, NC) and Mplus (version 6.1) [58].

## Results

We recruited 145 patients with DM who met the selection criteria and agreed to participate. Of this sample, 133 patients (91.72%) completed all the questionnaires without missing information. The sociodemographic and clinical characteristics of the sample are summarized in Table 1. The mean age was 62.74 years (SD, 12.18), 47.37% were women, 45.38% were obese and 70.68% had a disease duration of ≥ 10 years.

**Table 1:** Characteristics of the study participants

Variables	Diabetes mellitus (*n* = 133)
**Sociodemographic variables**	
**Age** (years), mean (SD)	62.74 (12.18)
**Age categorized** (years), n (%)	
≤49	17 (12.78)
50–59	32 (24.06)
60–69	45 (33.84)
≥70	39 (29.32)
**Gender**, women, n (%)	63 (47.37)
**BMI**, n (%)	
Non obese (< 30 kg/m^2^)	71 (54.62)
Obese (≥ 30 kg/m^2^)	59 (45.38)
**Employment status**, n (%)	
Active	40 (33.33)
Unemployed	13 (10.83)
Retired	67 (55.83)
**Level of education**, n (%)	
No studies	3 (2.61)
Primary studies	46 (40.00)
Secondary studies	22 (19.13)
High school diploma	22 (19.13)
Bachelor’s degree	22 (19.13)
**Clinical variables**	
**Disease duration** (years), mean (SD)	17.78 (10.54)
**Disease duration categorized**, n (%)	
< 10 years	39 (29.32)
≥ 10 years	94 (70.68)
**Smoking status**, n (%)	
Non-smoker	60 (46.15)
Ex-smoker	51 (39.23)
Smoker	19 (14.62)
**Regular physical activity**, n (%)	55 (44.00)
**Type of DM**, n (%)	
Type 1	24 (18.05)
Type 2	107 (80.45)
Mixed DM	2 (1.50)
**Insulin treatment**, n (%)	81 (61.36)
**Self-monitoring of blood glucose**, n (%)	104 (78.79)
**Glycemic control**, n (%)	
Poor control (HbA1c ≥ 7%)	74 (56.06)
Optimal control (HbA1c < 7%)	58 (43.61)
**Charlson Index**, mean (SD)	2.38 (1.67)
**Charlson Index**, n (%)	
1	50 (37.88)
2	38 (28.79)
> 2	44 (33.33)
**HRQOL questionnaires**	
**ADDQoL**, mean (SD)	
Present HRQOL	0.80 (0.84)
Diabetes influence on HRQOL	-1.68 (0.88)
Average weighted impact	-2.02 (1.57)
**HADS anxiety**, mean (SD)	6.06 (3.85)
**HADS anxiety level**, n (%)	
No (≤ 7)	90 (71.43)
Minor (8–10)	17 (13.49)
Moderate to severe (≥ 11)	19 (15.08)
**HADS depression**, mean (SD)	4.10 (3.21)
**HADS depression level**, n (%)	
No (≤ 7)	104 (82.54)
Minor (8–10)	13 (10.32)
Moderate to severe (≥ 11)	9 (7.14)
**SF-36 general health today**, mean (SD)	2.65 (0.81)
**SF-36 general health today**, n (%)	
Poor	8 (6.20)
Fair	46 (35.66)
Good	60 (46.51)
Very good	13 (10.08)
Excellent	2 (1.55)

SD, Standard deviation; BMI, Body Mass Index; HbA1c, glycosylated hemoglobin; HRQOL, Health Related Quality of Life; HADS, Hospital Anxiety and Depression Scale (each subscale ranges from 0 to 21, with a higher score indicating a higher level of mood disorder); ADDQoL, Audit on Diabetes-Dependent Quality of Life (Diabetes influence on HRQOL scored from − 3 as maximum negative impact to + 1 as maximum positive impact; the present HRQOL ranges from − 3 (extremely poor) to + 3 (excellent); and the average weighted impact (WI) ranges from − 9 to + 3, the lower the value of the average WI score, the worse the aspect of life); SF-36, 36-item Short-Form Health Survey; the SF-36 general health today ranges from 1 to 5, in which the higher the score, the better general health

**Fig. 1:**
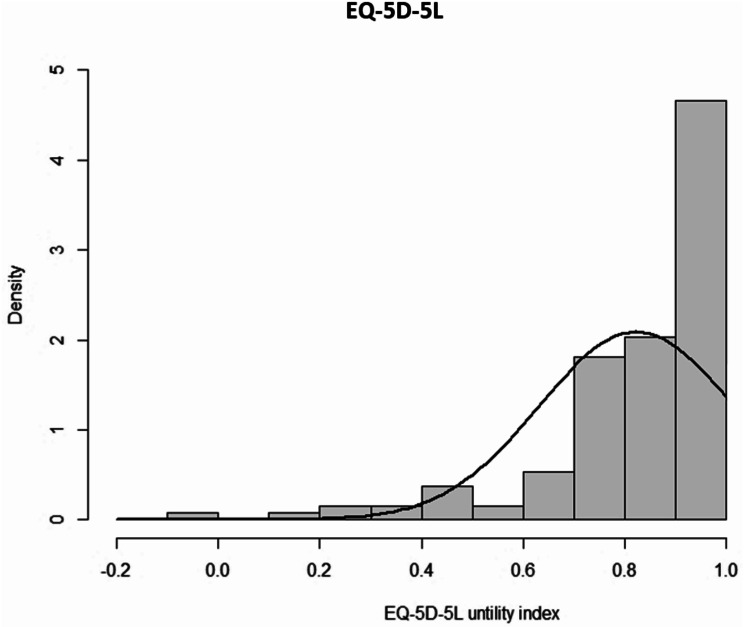
Distribution of the EQ-5D-5L utility index in the cohort of study. Density is represented in the histogram’s bars, while the black line indicates the normal distribution. The EQ-5D-5L utility index ranges from − 0.416 (worse than dead) to 1 (full health)

Table 2 lists the descriptive statistics of the EQ-5D-5L. The percentage of missing values ranged from 5.52 to 8.28%. The percentage of patients who had “no problems” in all EQ-5D-5L dimensions ranged from 45.86% for pain/discomfort to 88.72% for self-care. The EQ-VAS score showed no floor effect and minimal ceiling effect (1.54%). Then EQ-5D-5L utility index showed no floor effect, but a ceiling effect of 22.56%. The distribution of the utility index is shown in Fig. 1.

**Table 2:** Descriptive statistics of the EQ-5D-5L dimensions (*n* = 133)

EQ-5D-5L	Completion^*^	EQ-5D-5L dimensions’s response options				
	*n* (%) of missing	No problems *n* (%)	Slight problems *n* (%)	Moderate problems *n* (%)	Severe problems *n* (%)	Unable to / extreme problems *n* (%)
Mobility	8 (5.5)	73 (54.89)	30 (22.56)	21 (15.79)	9 (6.77)	0 (0)
Self-care	10 (6.9)	118 (88.72)	8 (6.02)	5 (3.76)	2 (1.50)	0 (0)
Usual activities	10 (6.9)	93 (69.92)	20 (15.04)	15 (11.28)	3 (2.26)	2 (1.50)
Pain/discomfort	9 (6.2)	61 (45.86)	42 (31.58)	19 (14.29)	10 (7.52)	1 (0.75)
Anxiety/depression	12 (8.3)	84 (63.16)	31 (23.31)	14 (10.53)	4 (3.01)	0 (0)
		**EQ-5D-5L scores**				
		**Mean (SD)**	**Median (IQR)**	**Range (min, max)**	**Floor, %**	**Ceiling, %**
EQ-5D-5L utility index	12 (8.3)	0.823 (0.191)	0.872 (0.756 − 0.922)	(-0.09, 1)	0	22.56
EQ-VAS	7 (4.8)	69.56 (19.12)	70 (60 − 85)	(10, 100)	0	1.54

^*^The completion n (%) of missing are calculated with the total of 145 patients

SD: Standard deviation; IQR: interquartile range

EQ-5D-5L utility index score range from − 0.416 to 1, with higher score indicating better HRQOL; EQ-VAS scale range from 0 (worst imaginable health) to 100 (best imaginable health)

### Reliabililty

Cronbach’s alpha was 0.78, being higher than the threshold of 0.70. The inter-item correlations ranged from 0.31 to 0.61, except for the correlation of the anxiety/depression dimension with the rest, being lower (between 0.05 and 0.27). The item-total correlations ranged from 0.21 to 0.68 (Table 3).

**Table 3:** Exploratory and confirmatory factor analysis, item response theory parameter estimates from graded response model for the EQ-5D-5L questionnaire, and item-scale correlation (*n* = 133)

Items	Exploratory factor analysis	Confirmatory factor analysis	Graded response model				Item-scale correlation^*^
	Factor loading	Factor loading	*α*	*β*_*1*_	*β*_*2*_	*β*_*3*_	ρ
EQ1. Mobility	0.84	0.79	1.49 (0.24)	0.18 (0.17)	1.28 (0.22)	2.78 (0.37)	0.52
EQ2. Self-care	0.91	0.70	1.78 (0.36)	2.37 (0.37)	3.35 (0.47)	5.04 (0.96)	0.43
EQ3.Usual activities	0.90	0.80	2.27 (0.41)	1.23 (0.34)	2.60 (0.49)	4.67 (0.84)	0.68
EQ4. Pain/discomfort	0.68	0.63	0.93 (0.16)	-0.17 (0.14)	1.03 (0.17)	1.98 (0.21)	0.48
EQ5. Anxiety/depression	0.36	0.40	0.37 (0.13)	0.32 (0.12)	1.15 (0.15)	2.04 (0.23)	0.21
% variance explained	64.82%						
χ^2^ (df)		6.15 (4)					
RMSEA (90% CI)		0.064 (0–0.157)					
CFI		0.989					
NNFI		0.973					

df = degrees of freedom; RMSEA = root mean square error of approximation; CI: confidence interval; CFI = comparative fit index; NNFI = Non-normed fit index; *α* = slope parameter estimate from the graded response model; *β*_1_, *β*_2_, and *β*_3_ = threshold parameters from the graded response model. Standard error estimates for each parameter estimate are listed in parentheses

^*^Item-scale correlation correcting for overlap calculated by Spearman correlation coefficient

### Item functioning

Regarding unidimensionality, the EFA (Table 3) yielded factor loadings between 0.36 and 0.91, exceeding the benchmark of 0.40, except for the anxiety/depression item. The percentage of variance explained by the factor was 64.82%. The ratio of the eigenvalues of the first and second unrotated factors was 12.67:1, exceeding the benchmark of 3:1. The results of the CFA showed adequate fit indices (Table 3): (a) the RMSEA was 0.064, less than 0.08; and (b) the CFI and NNFI were 0.989 and 0.973, respectively. Factor loadings ranged from 0.40 to 0.80 and were all significant (*p* < 0.001).

Table 3 lists the slope (*α*) and threshold (*β*) parameters estimated from the GRM. Regarding *α*, all values were acceptable and large, except for the anxiety/depression item. The highest *α* was obtained for item 3 (usual activities), with a value exceeding 2, followed by items 2 (self-care) and 1 (mobility), with values somewhat lower, but still high (between 1.5 and 2). Item 4 (pain/discomfort) showed a slightly lower discriminatory power (*α* = 0.93), but not negligible. Finally, item 5 (anxiety/depression) had negligible discriminatory power (*α* = 0.37). Regarding *β*, item 3 (usual activities) covers the largest range of the trait, with values of around 1 *SD* from the mean in *β*_1_ to around 4.5 *SD* in *β*_3_, indicating that this item is more apparent at higher levels of severity. Item 2 (self-care) also covers quite a large range of the trait, but is most apparent at higher levels of severity, with values from 2.5 *SD* from the mean in *β*_1_ to 5 *SD* in *β*_3_. Item 1 (mobility) covers a similar range of the trait but is most apparent at medium levels of severity. Finally, items 4 (pain/discomfort) and 5 (anxiety/depression) cover a narrower range of the trait, with values of around − 0.25 *SD* to 0.25 *SD* from the mean in *β*_1_, respectively, to around 2 *SD* in *β*_3_, being more apparent at lower levels of severity.

**Fig. 2:**
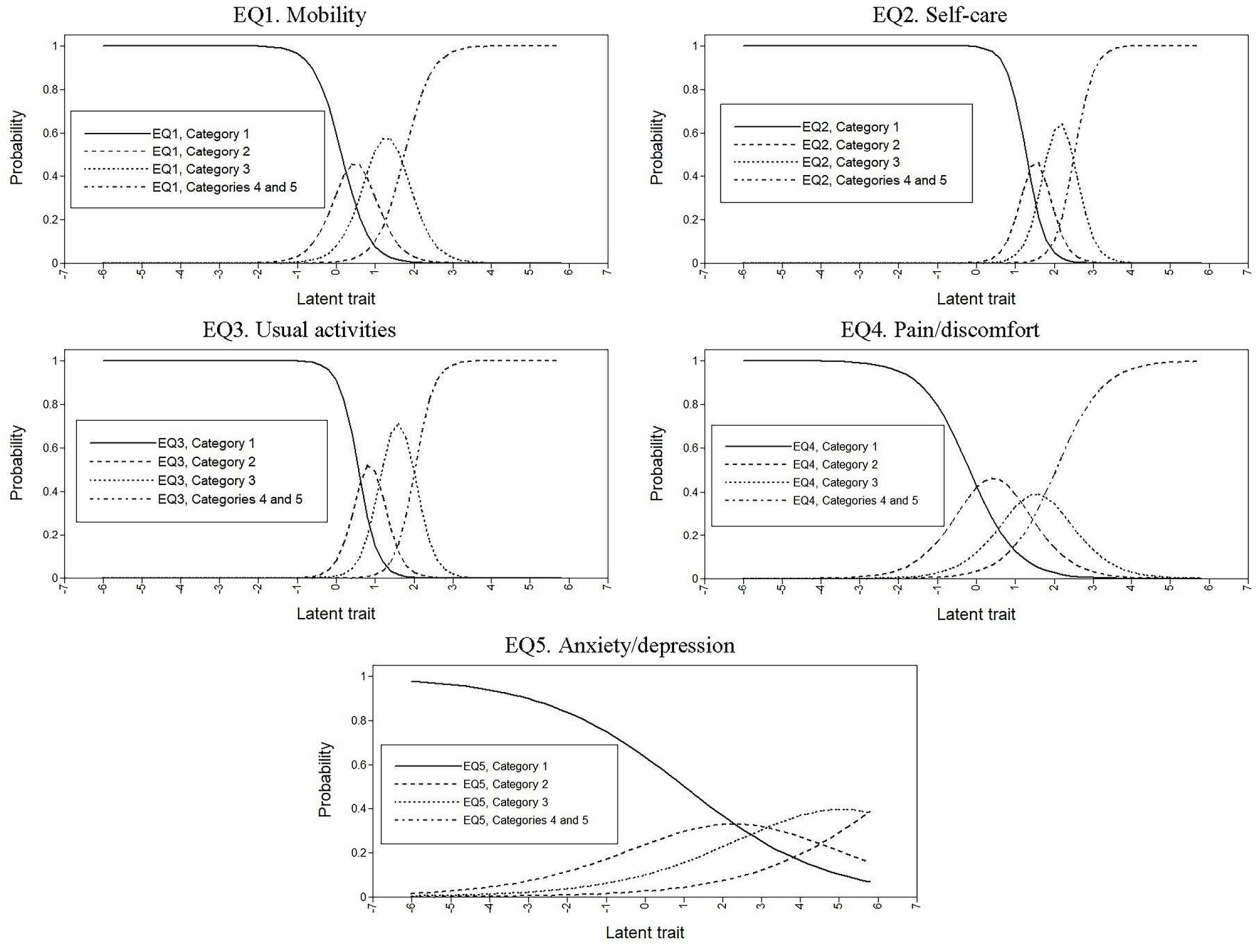
Category response curves for the EQ-5D-5L items. The x-axis represents the latent trait and the y-axis represents the probability of a positive response to each item’s response option. Category 1 refers to “No problems”, category 2 to “Slight problems”, category 3 to “Moderate problems”, category 4 to “Severe problems” and category 5 to “Unable to/Extreme problems”

**Fig. 3:**
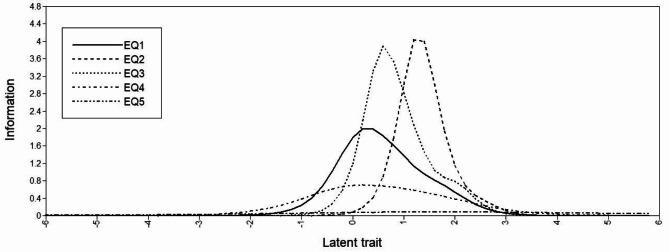
Q

The CRCs for all items showed a shift to higher trait levels as the level of the response increased, but not very notable for item 5 (anxiety/depression) (Fig. 2). Regarding the IICs (Fig. 3), we can see that values for items 2 (self-care) and 3 (usual activities) were relatively high across the trait spectrum compared with the rest, and in particular compared with item 5 (anxiety/depression). Regarding the model fit, the residuals of all categories of all items were not significantly different from 0, and there were no significant differences between the observed and expected likelihoods in any of the five items, demonstrating the good fit.

### Convergent and discriminant validity

The ADDQoL present HRQOL dimension showed high correlations with all EQ-5D-5L dimensions, except in the cases of self-care (*ρ*=-0.42) and anxiety/depression (*ρ*=-0.27); and a small correlation with the influence of diabetes on HRQOL item and the average WI dimension. Further, we found a high correlation between the two HADS dimensions and the EQ-5D-5L anxiety/depression domain, and between HADS depression dimension and the EQ-5D-5L utility index, and a lower correlation with the other four dimensions and the EQ-VAS. The general health item presented a high correlation with the pain/discomfort dimension, EQ-5D-5L index and EQ-VAS (*ρ*=-0.58, 0.59 and 0.58, respectively), small correlation with anxiety/depression (*ρ*=-0.19), and moderate correlation with the rest of dimensions (Table 4).

**Table 4:** Convergent and divergent validity (*n* = 133)

	Mobility	Self-care	Usual activities	Pain / discomfort	Anxiety / depression	EQ-5D-5L utility index	EQ-VAS
**ADDQoL**							
Present HRQOL	-0.50	-0.42	-0.58	-0.54	-0.27	0.62	0.54
Diabetes influence on HRQOL	0.08	-0.01	0.01	-0.04	0.08	-0.04	-0.14
Average weighted impact	-0.23	-0.07	-0.14	-0.18	-0.13	0.27	0.10
**HADS**							
Anxiety	0.04	0.15	0.23	0.34	0.51	-0.37	-0.26
Depression	0.35	0.33	0.47	0.48	0.50	-0.61	-0.46
**SF-36 general health today**	-0.49	-0.31	-0.40	-0.58	-0.19	0.59	0.58

HADS = Hospital Anxiety and Depression Scale; ADDQoL = Audit on Diabetes-Dependent Quality of Life

Data are given as Spearman’s correlation coefficient

The scores for the ADDQoL subscales range from − 3 to 3 in present HRQOL item, with higher scores indicating better health status; from − 3 to 1 in the diabetes influence on HRQOL item, with higher scores indicating more positive impact; and from − 9 to 3, with higher scores indicating more positive impact in diabetes. The scores for the HADS ranges from 0 to 21, with higher score indicating a higher level of mood disorder. Items concerning the five dimensions in EQ-5D-5L (mobility, self-care, usual activities, pain/discomfort and anxiety/depression) are rated from 1 (no problems) to 5 (unable to/extreme problems). The scores for the EQ-5D-5L utility index ranges from − 0.416 to 1, with higher score indicating better HRQOL, and EQ-VAS scale range from 0 (worst imaginable health) to 100 (best imaginable health); SF-36, 36-item Short-Form Health Survey; the SF-36 general health today ranges from 1 to 5, in which the higher the score, the better general health

### Known-groups validity

There were significant differences in the EQ-5D-5L index between subgroups defined by age, gender, BMI, regular physical activity, disease duration, HbA1c, general health today rating, and the HADS anxiety and depression level. EQ-5D-5L utility index scores were significantly lower in patients who were older, women, obese, did not do regular physical activity, with disease duration of ≥ 10 years, poor glycemic control, poorer general health status, and higher anxiety or depression level. In EQ-VAS, there were significant differences only between subgroups defined by age, sex, regular physical activity, disease duration, general health today, and HADS anxiety and depression level (Table 5).

**Table 5:** Known-groups validity of the EQ-5D-5L

	EQ-5D-5L utility index		EQ-VAS	
	*N*	Mean (SD)	*N*	Mean (SD)
**Age** (years)				
≤49^a^	17	0.839 (0.261)	17	74.41 (17.93)
50–59^b^	32	0.901 (0.104)^d^	32	75.13 (15.10)
60–69^c^	45	0.816 (0.172)	43	69.05 (22.92)
≥ 70^d^	39	0.759 (0.213)^b^	38	63.29 (16.41)
*p-*value		0.018		0.046
**Gender**				
Men	70	0.880 (0.134)	70	73.63 (16.65)
Women	63	0.759 (0.222)	63	64.82 (20.79)
*p-*value		0.0002		0.0137
**BMI**				
Obese (≥ 30 kg/m^2^)	59	0.775 (0.227)	57	67.02 (20.59)
Non obese (< 30 kg/m^2^)	71	0.870 (0.137)	70	72.11 (17.31)
*p-*value		0.004		0.132
**Regular physical activity**				
Yes	55	0.906 (0.102)	53	78.28 (14.31)
No	70	0.761 (0.223)	69	62.68 (20.32)
*p-*value		< 0.001		< 0.001
**Disease duration (years)**				
< 10	39	0.896 (0.100)	39	76.67 (16.20)
≥ 10	94	0.792 (0.211)	91	66.52 (19.54)
*p-*value		0.005		0.004
**Glycemic control**				
Poor control (HbA1c ≥ 7%)	74	0.781 (0.227)	72	67.28 (19.61)
Optimal control (HbA1c < 7%)	58	0.875 (0.119)	57	72.79 (18.17)
*p-*value		0.005		0.104
**Type of DM**				
Type 1	24	0.854 (0.225)	24	73.71 (20.03)
Type 2	107	0.816 (0.185)	104	68.79 (19.02)
*p-*value		0.150		0.179
**Health today (SF-36)**				
Poor^a^	8	0.430 (0.353)^bcd^	8	32.5 (14.64)^bcd^
Fair^b^	46	0.752 (0.167)^acd^	45	63.11 (16.66)^acd^
Good^c^	60	0.899 (0.089)^ab^	59	75.24 (12.81)^abd^
Very good + Excellent^d^	15	0.934 (0.120)^ab^	14	87.86 (17.18)^abc^
*p-*value		< 0.001		< 0.001
**HADS anxiety level**				
No (≤ 7)^a^	90	0.872 (0.138)^bc^	89	72.74 (17.99)
Minor (8–10)^b^	17	0.755 (0.180)^a^	16	63.75 (20.29)
Moderate to severe (≥ 11)^c^	19	0.684 (0.243)^a^	19	62.58 (19.17)
*p-*value		< 0.001		0.0376
**HADS depression level**				
No (≤ 7)^a^	104	0.870 (0.128)^bc^	103	73.05 (16.80)^c^
Minor (8–10)^b^	13	0.614 (0.301)^a^	12	60.83 (25.30)
Moderate to severe (≥ 11)^c^	9	0.580 (0.200)^a^	9	46.11 (11.40)^a^
*p-*value		< 0.001		< 0.001

SD: Standard deviation; BMI, Body Mass Index; HbA1c, Glycosylated hemoglobin; DM: Diabetes; SF-36, 36-item Short-Form Health Survey; HADS = Hospital Anxiety and Depression Scale

The score for the EQ-5D-5L utility index range from − 0.416 to 1, and the score for the EQ-VAS range from 0 to 100, with higher scores indicating better health status

^a, b,c, d,e^Superscript letters indicated differences among the subgroups by Scheffe’s test for multiple comparisons at *p* < 0.05

## Discussion

The results of this prospective study with a cohort of DM patients seen in two hospitals in the Basque Country (Spain), combining both classical psychometric and IRT approaches, support the validity and reliability of the EQ-5D-5L at item and scale levels. To the best of our knowledge, this is the first study assessing the psychometric properties of the Spanish EQ-5D-5L in patients with DM.

Regarding completion of the EQ-5D-5L, the missing data rate of between 5.52% and 8.28% was quite high [27, 30]. This is likely attributable to the combined length of the questionnaires used (EQ-5D-5L, ADDQoL, and HADS), and hence, completion of the questionnaires may have been a considerable burden for participants.

As stated, the EQ-5D-5L was developed to address the main limitations of the EQ-5D-3L, in particular, the ceiling effect of the utility index [18, 27]. However, in some studies the ceiling effect has been found to remain high when the EQ-5D-5L is used in DM, with ceiling effects greater than 15% [27, 30–32, 59], as in our study (22.56%). This is probably because our respondents were likely to perceive themselves as healthy, consistent with their EQ-VAS median score of 70. Nevertheless, other studies in DM patients have shown small ceiling effects in the 5L utility index [26, 29]. Further, in our sample, the ceiling effect of the EQ-5D-5L at the item level (those responding “no problems”) was also high. In any case, considering that items are categorical, despite having five categories instead of three, it is easy for floor or ceiling effects to occur. Similar results have been found in other studies [26–32, 59–63], being the highest ceiling effect in the self-care dimension.

The Cronbach’s alpha exceeded the threshold of 0.70 [45], indicating that 5L version maintained good internal consistency. This result agrees with those found by Abedini et al. [28].

The item functioning of the EQ-5D-5L was studied using IRT, and more specifically, the graded response model (GRM), which is a two-parameters IRT model [52]. Given that the factor loading of the anxiety/depression item is much lower than the others and therefore contributes less to the trait, we need to apply a two-parameter IRT model allowing different discriminatory ability to each EQ-5D-5L item. The results of the factor analyses support the unidimensionality and adequacy of applying IRT. The results of the IRT indicated that the data were well fitted by the GRM. The item with the highest discriminatory power was usual activities, followed by self-care dimension, and mobility dimension. Likewise, *α* parameters of the GRM are consistent with the EFA and CFA factor loadings, the anxiety/depression dimension being the item with the lowest discriminatory power and usual activities with the highest. Moreover, regarding the range of the trait, the usual activities dimension covers the largest range, and it is more apparent at higher levels of severity in these patients. On the other hand, the pain/discomfort and anxiety/depression items cover a narrower range of the trait and are more apparent at lower levels of severity. These results make sense in that these items have shown not to be particularly useful in this disease in which patients do not have high levels of severity.

At scale-level, the convergent and discriminant validity was also demonstrated confirming our hypotheses. Nonetheless, the correlation of EQ-5D-5L self-care and anxiety/depression dimension with the ADDQoL present HRQOL dimension was lower than expected. This could be due to these EQ-5D-5L dimensions reflect more psychological aspects of HRQOL, while perhaps, ADDQoL present HRQOL dimension reflects more physical aspects. Further, we found a slightly higher correlation between the SF-36 general health and pain/discomfort dimension, perhaps because pain is often closely related to self-perceive HRQOL. Similarly good convergent validity has also been reported in other studies of DM. Jankowska et al. [29] also confirmed the convergent validity of the EQ-5D-5L in respondents with self-reported diabetes from a representative general population survey, examining the association between this instrument and the SF-12. Pattanaphesaj et al. [30] also found satisfactory results for convergent validity of the EQ-5D-5L dimensions studying the correlations with the SF-36.

The EQ-5D-5L also showed excellent known-groups validity, with statistically significant differences according to different characteristics. As other previous studies [28, 29, 31, 57], we have also found statistically significant lower EQ-5D-5L utility index scores in patients who were older, women, obese, did not do regular physical activity, have a higher disease duration, poor glycemic control (HbA1c ≥ 7%), had a poorer SF-36 “health today rating”, and higher level of anxiety and depression. Therefore, the 5L version seems to have the ability to discriminate between different levels of health. This validity has also been shown in the general population [63–66], as well as in specific diseases other than DM [21, 22, 27, 67].

In addition to those mentioned above, this study has various limitations. First, the recruitment period is almost 10 years ago. However, it is not usual to update the psychometric properties of instruments every few years, because analysis regarding their underlying structure, reliability, relationship with other instruments or the known-groups validity are valid regardless to the recruitment timing. Then, our findings are of a timeless nature, also, useful and reproducible in the modern-day context. Second, we were unable to explore differences between patients who agreed to participate in the study and those who were invited but did not accept. Thus, we were unable to assess the representativeness of the sample. Nonetheless, we believe that the patients included in this study cover the different levels of severity commonly seen in daily practice. To complete the validity study, the questionnaire’s responsiveness would need to be analyzed, and although we studied the internal consistency, the reliability study should be complemented with a test-retest study. Further, the sample size is somewhat small for IRT analysis, and although we have used a Bayesian estimator to minimize this limitation, the results should be treated with caution. As the sample was not large enough to allow IRT analysis by groups, it was not possible to investigate differential item functioning, which occurs when different groups within the sample respond in different ways to an individual item. Another limitation could be the use of patient-generated outcome measures, such as the ADDQoL questionnaire, since they do not provide a form of standardization needed for comparison of results. However, we are using the ADDQoL only to study the convergent validity of the EQ-5D-5L, and despite this limitation, these measures are adequate in the development of other instruments [68]. Finally, although serum Hb1Ac is still the gold standard measure for the glycemic control, advances over the last decade have brought to light its problematics regarding the diabetes monitoring and the association with the risk of complications in certain clinical instances [69, 70]. Besides, Hb1Ac has been reported not to be surrogate for HRQOL when measured by other specific questionnaires [71]. However, our results are consistent with others demonstrating the relationship with the EQ-5D-5L [28, 57]. We recommend including additional diabetes monitoring variables in future researches so evaluation of HRQOL in DM can be adapted to the new management procedures.

## Conclusions

Despite not all measurement properties have been tested in the current study, the results provide evidence of the validity of the EQ-5D-5L in patients with DM in Spain. The study provides evidence for clinicians and researchers concerning the appropriateness use of this generic preference-based measure for assessing HRQOL in patients with DM, and for use in economic evaluation. In conclusion, our prospective study of the psychometric properties of the Spanish version of the EQ-5D-5L in patients with DM, combining both classical test theory and IRT, provides evidence of its adequate item functioning, reliability and validity in this population.

## Data Availability

The datasets used and/or analysed during the current study are available from the corresponding author on reasonable request.

## Abbreviations

ADDQoL: Audit on diabetes-dependent quality of life
BMI: Body mass index
CFA: Confirmatory factor analysis
CFI: Comparative fit index
CRC: Category response curve
DM: Diabetes mellitus
EFA: Exploratory factor analysis
EQ-VAS: EQ-visual analogue scale
GRM: Graded response model
HADS: Hospital anxiety and depression scale
HbA1c: Glycosylated blood hemoglobin
HRQOL: Health related quality of life
IIC: Item information curve
IRT: Item response theory
NNFI: Non-normed fit index
QALY: Quality-adjusted life years
RMSEA: Root mean square error of approximation
SD: Standard deviation
WI: Weighted impact
